# Bee Venom: Composition and Anticancer Properties

**DOI:** 10.3390/toxins16030117

**Published:** 2024-02-29

**Authors:** Goran Gajski, Elina Leonova, Nikolajs Sjakste

**Affiliations:** 1 Division of Toxicology, Institute for Medical Research and Occupational Health, 10000 Zagreb, Croatia; 2 Department of Medical Biochemistry, Faculty of Medicine, University of Latvia, 1004 Riga, Latvia; 3 Genetics and Bioinformatics, Institute of Biology, University of Latvia, 1004 Riga, Latvia

**Keywords:** natural products, apitherapy, apitoxin, bee venom, melittin, phospholipase A_2_, anticancer properties, therapeutic application

## Abstract

Among the various natural compounds used in alternative and Oriental medicine, toxins isolated from different organisms have had their application for many years, and *Apis mellifera* venom has been studied the most extensively. Numerous studies dealing with the positive assets of bee venom (BV) indicated its beneficial properties. The usage of bee products to prevent the occurrence of diseases and for their treatment is often referred to as apitherapy and is based mainly on the experience of the traditional system of medical practice in diverse ethnic communities. Today, a large number of studies are focused on the antitumor effects of BV, which are mainly attributed to its basic polypeptide melittin (MEL). Previous studies have indicated that BV and its major constituent MEL cause a strong toxic effect on different cancer cells, such as liver, lung, bladder, kidney, prostate, breast, and leukemia cells, while a less pronounced effect was observed in normal non-target cells. Their proposed mechanisms of action, such as the effect on proliferation and growth inhibition, cell cycle alterations, and induction of cell death through several cancer cell death mechanisms, are associated with the activation of phospholipase A_2_ (PLA_2_), caspases, and matrix metalloproteinases that destroy cancer cells. Numerous cellular effects of BV and MEL need to be elucidated on the molecular level, while the key issue has to do with the trigger of the apoptotic cascade. Apoptosis could be either a consequence of the plasmatic membrane fenestration or the result of the direct interaction of the BV components with pro-apoptotic and anti-apoptotic factors. The interaction of BV peptides and enzymes with the plasma membrane is a crucial step in the whole process. However, before its possible application as a remedy, it is crucial to identify the correct route of exposure and dosage of BV and MEL for potential therapeutic use as well as potential side effects on normal cells and tissues to avoid any possible adverse event.

## 1. Introduction

**Figure 1:**
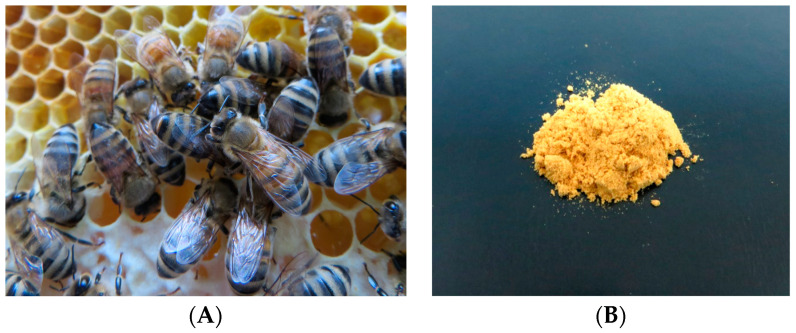
European honey bee *Apis mellifera* (**A**) and lyophilized bee venom (**B**).

Regardless of the significant developments in modern medicine, pharmaceuticals resulting from plant and animal species continuously make important contributions to health in terms of the prevention and treatment of numerous diseases [1,2,3,4,5]. Many treatments that are frequently used in Western countries come from Asia, and their popularity is increasing rapidly [6,7,8,9]. Animal venoms, especially those from insects, have historical usage in scientific research, and are used today as a source of various products and drugs with potential medical applications [10,11,12,13,14,15]. Among the several natural compounds that have found their place in Oriental and alternative medicine, toxins isolated from a large number of organisms have had their application for many years, the most important being bee venom (BV) isolated from *Apis mellifera* (Figure 1A) [3,12,16,17]. Numerous studies speak in favor of the positive properties of BV, which include its radioprotective [18,19,20], antimutagenic [21,22], anti-inflammatory [23,24], antinociceptive [24,25], and antimicrobial [26,27] properties.

In recent decades, the number of cancer patients and their mortality rate have increased, thereby creating enormous health as well as economic problems, especially in the aging population [28]. The increasing incidence of tumors leads to the need to create novel drugs and strategies for the prevention and treatment of this particular disease. Therefore, anticancer research and exploration of different options for cancer treatment are some of the key tasks of modern science [29,30,31]. The efforts of modern medicine and science to discover remedies for this disease have not been completely successful, and although today prescribed therapies such as surgery, radiotherapy, and chemotherapy help patients to control the disease, the result is still lethal for many of them. Hence, recently we have seen an increased reliance on alternative treatments for these diseases, based mostly on using natural compounds of plant and animal origin in therapies against tumors [32,33,34,35,36,37].

Different animal toxins isolated from spiders, scorpions, snakes, snails, sea urchins, and corals can kill cancer cells [1,12,15,38,39,40,41,42]. Furthermore, in recent years growing importance has been given to bee products, especially BV, which is used for a variety of medicinal purposes [12,16,21,22,35]. The use of bee products for preventing and treating many different diseases is referred to as apitherapy. The use of natural products and their active ingredients in the prevention and treatment of chronic diseases is largely based on the experience of the traditional medical system found in different ethnic communities and epidemiological data of the relationship between diet and disease [12,21,35,36]. Although *Apis mellifera* venom has been studied the most extensively, there are several other *Apis* species, namely, *Apis cerana* and *Apis florea*, whose venom and/or peptides have also shown promising pharmacological effects and anticancer properties [43,44].

Interest in the medicinal properties of bee products that have been known for millennia, as well as their potential anticancer effects, increased in the last 30 years, and the composition of bee products is being researched for their possible biological activities with modern methodological approaches. Nowadays, there are more than 20,000 species of bees in the world, and people’s interest is largely related to the medicinal properties of bee products such as propolis, honey, beeswax, pollen, and royal jelly, as well as BV [35,45,46]. In recent years, numerous studies have also discussed the anticancer properties of BV and its components. Hence, recent studies point to several mechanisms of toxicity of this natural compound towards various cancer cells that include changes in the cell cycle, effects on cell survival and proliferation, and the induction of both apoptosis and necrosis as the cell death mechanism [3,12,16,21,47]. Although there are numerous animal venoms and components that often show good results towards cancer cells, there are always open questions regarding their potential toxicity to normal non-target cells and tissues, which is one of the biggest obstacles to applying such natural products as medications. Therefore, in this review we will summarize the composition of BV and its anticancer properties, as well as possible ways to overcome obstacles to its usage as a therapeutic modality.

## 2. Bee Venom

BV is a secretion from the venom gland of bees that is used to defend bee colonies from the enemy and as a warning signal. It is assumed that after a bee sting, a volatile part of the venom evaporates and serves as a certain alarm to other bees regarding the presence of an enemy. BV is positioned in the bee’s abdominal cavity, which is on one side connected with a venom gland that secretes venom, while on the other side, it is connected by a small canal that leads to the stinger. The venom gland produces venom that pours into the venom gland. A few weeks after metamorphosis, bees have the highest amount of venom in the gland. While flying and collecting nectar, the level of venom gradually decreases. Upon a sting, a bee injects 50 to 140 µg of venom. The bee stinger consists of two parallel needles that have hooks for deeper penetration and attachment and they release the venom through a channel. The stinger is located in the abdomen and is released upon stinging. Due to its specific build, after a sting, bees are not able to remove it and the stinger itself along with the venom gland remains in the skin of a vertebrate. The muscles that hold the structure break and the bee dies [48,49,50,51,52,53,54].

In mammals, BV causes toxic effects throughout the whole body, especially on the cardiovascular and nervous systems. Because of its diverse composition, the venom has various effects on multiple organs. In its effect, BV is very similar to snake venom, but the amount of venom that is released during a bee sting is much lower compared to a snake bite. The venom itself causes the degradation of blood cells, reduces the ability of blood clotting, and increases the permeability of blood vessels as evidenced by swelling and bleeding in internal organs. In humans, BV causes an inflammatory reaction manifested by swelling, redness, and pain at the injection site. The most dangerous are stings in the mouth, tongue, or eyeball. A large amount of BV can also be deadly in certain cases and death can occur due to the bronchial spasm that arises due to the paralysis of the brain center responsible for breathing. BV in the body causes an immune system reaction which, in hypersensitive individuals, causes allergic reactions that can be very dangerous and can also induce death [55,56,57,58,59,60,61,62].

For scientific purposes, BV is collected with a special device made of glass panels and wires emitting a low-voltage current from 18 to 22 V. This device is placed at the entrance to the bee hive. Under the influence of an electric field, irritated bees secrete their venom directly to the slide from which venom is scraped after drying. BV is a thick liquid with a characteristic odor resembling honey and a bitter sour taste. Lyophilized BV that is dried without the liquid phase is a volatile, light gray to grayish-yellow powder (Figure 1B). As such, BV is kept in airtight containers at −20 °C according to the manufacturer’s instructions [63,64,65,66].

## 3. The Composition of Bee Venom

**Figure 2:**
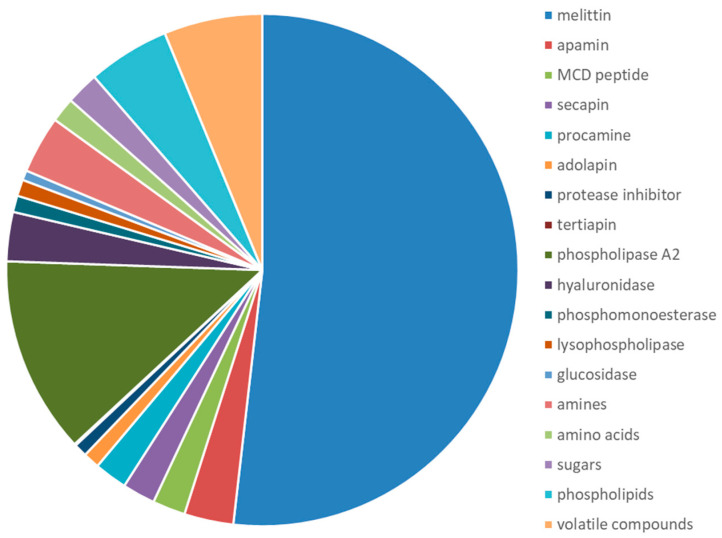
Composition of dry bee venom.

The main component of BV is water which makes up about 88% of the venom itself. Other dry parts of BV comprise the peptides melittin (MEL), apamin, secapin, procamine A and B, adolapin, tertiapin, and mast cell degranulating (MCD) peptide. The dominant enzymes in BV are phospholipase A_2_ (PLA_2_) and at a lower rate phospholipase B (PLB), hyaluronidase, acid phosphomonoesterases, lysophospholipase, and α-glucosidase. BV is also composed of several physiologically active amines and neurotransmitters (histamine, dopamine, and noradrenalin), glucose and fructose, phospholipids, amino acids, and higher amounts of mineral substances (Figure 2 and Table 1). Nearly all of these components which are contained in BV have effects on many cell systems to some extent. The three most abundant peptide components of BV are MEL, apamin, and MCD peptide [12,15,21,49,67,68,69,70,71]. It has to be pointed out that the composition of bee venom is subject to various factors such as the region and time of year when the venom is collected [47,72].

### 3.1. Melittin

**Figure 3:**
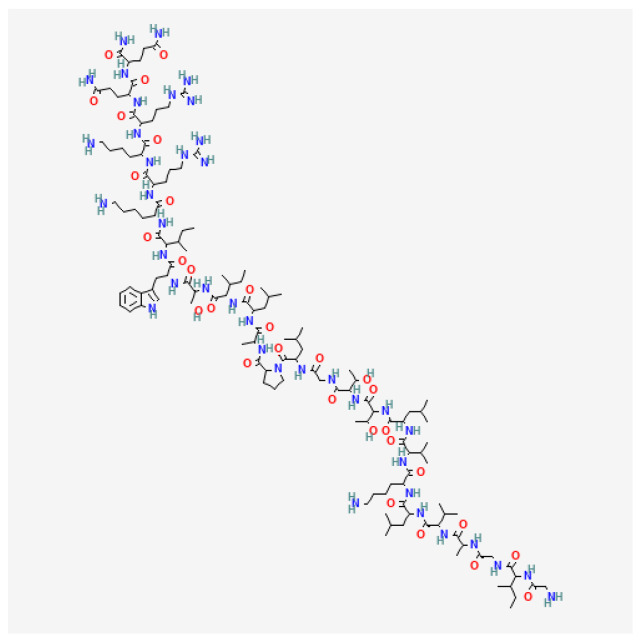
Structure of melittin (Gly-Ile-Gly-Ala-Val-Leu-Lys-Val-Leu-Thr-Thr-Gly-Leu-Pro-Ala-Leu-Ile-Ser-Trp-Ile-Lys-Arg-Lys-Arg-Gln-Gln). National Center for Biotechnology Information. PubChem Compound Summary for CID 16129627, Melitten. https://pubchem.ncbi.nlm.nih.gov/compound/Melitten (Accessed on 15 January 2024).

MEL is a major component and toxin of BV comprising about 50% of the dry venom based on literature data. MEL is a basic peptide consisting of the 26 known amino acid sequences with a molecular weight of 2847.5 Da (Figure 3). The peptide amino acid sequence is Gly-Ile-Gly-Ala-Val-Leu-Lys-Val-Leu-Thr-Thr-Gly-Leu-Pro-Ala-Leu-Ile-Ser-Trp-Ile-Lys-Arg-Lys-Arg-Gln-Gln [73,74,75]. MEL is an amphoteric molecule because of the specific arrangement of amino acids in its chain. At the N-terminus (positions 1 to 20), there are non-polar, hydrophobic, and neutral amino acids, whereas at the C-terminus (positions 21 to 26) there are hydrophilic and basic amino acids. This amino acid arrangement gives MEL amphipathic properties. MEL is regarded as a natural detergent with high surface and membrane tension. Though it is soluble as a tetramer or monomer, MEL can easily incorporate in the membrane and form ion pores that lead to disorder in the phospholipid bilayer structure. MEL tetramers cause the depolarization of nerve endings and trigger pain [76,77,78]. MEL may also enhance the activity of PLA_2_ and thus also affect cells [79,80]. Each MEL chain has two α-helical segments forming a bent rod. MEL occurs as a tetramer at a concentration present in the venom gland and as a monomer at a minimal concentration necessary for cell lysis [73,74,75,81]. These structural features may have a crucial role in its cytotoxic properties.

So far, data suggest that MEL is toxic to hematopoietic cells including lymphocytes, erythrocytes, and thymocytes, as well as to intestinal cells [15,82,83,84,85,86]. Furthermore, MEL may impact several metabolic functions of cells by disturbing the plasma membrane and causing changes in the enzymatic system, whereas its lytic activity is mainly related to the possibility of integrating into the cell membrane phospholipid bilayer [87,88].

### 3.2. Apamin

**Figure 4:**
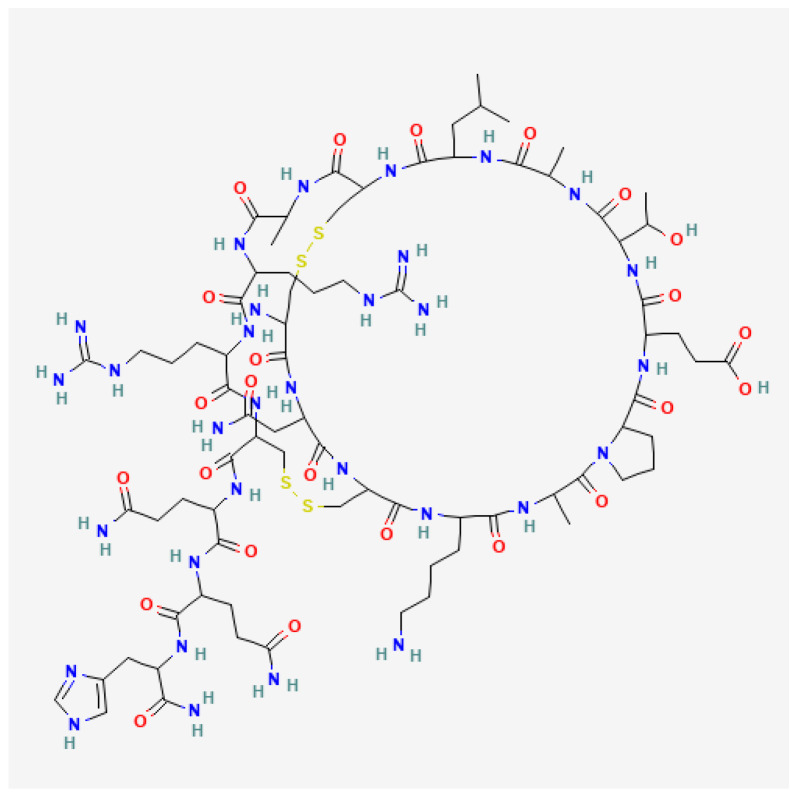
Structure of apamin (Cys-Tyr-Cys-Lys-Ala-Pro-Glu-Thr-Ala-Leu-Cys-Ala-Arg-Arg-Cys-Gln-Gln-His). National Center for Biotechnology Information. PubChem Compound Summary for CID 44134548, Apamine. https://pubchem.ncbi.nlm.nih.gov/compound/Apamine (Accessed on 15 January 2024).

Apamin as the BV neurotoxin is a rather small basic peptide consisting of 18 amino acids with two disulfide bridges, which makes the structure extremely solid with a molecular weight of 2027.3 Da (Figure 4). The apamin amino acid sequence is Cys-Tyr-Cys-Lys-Ala-Pro-Glu-Thr-Ala-Leu-Cys-Ala-Arg-Arg-Cys-Gln-Gln-His [89,90]. The peptide causes neurotoxic effects in mammals’ spinal cord, which leads to muscle spasms. Moreover, apamin owns a selective inhibitory action on calcium-dependent potassium channels [91,92,93].

### 3.3. Mast Cell Degranulating (MCD) Peptide

**Figure 5:**
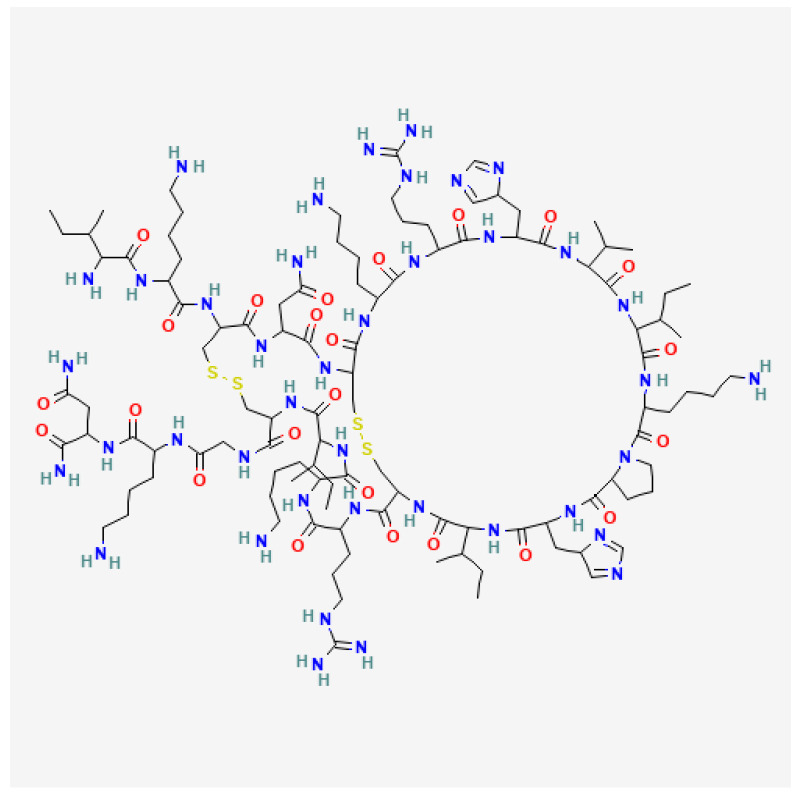
Structure of mast cell degranulating (MCD) peptide (Ile-Lys-Cys-Asn-Cys-Lys-Arg-His-Val-Ile-Lys-Pro-His-Ile-Cys-Arg-Lys-Ile-Cys-Gly-Lys-Asn). National Center for Biotechnology Information. PubChem Compound Summary for CID 16132290, Mast cell degranulating peptide. https://pubchem.ncbi.nlm.nih.gov/compound/Mast-cell-degranulating-peptide (Accessed on 15 January 2024).

The MCD peptide is a basic peptide consisting of 22 amino acids with two disulfide bridges and a molecular weight of 2587.2 Da (Figure 5). The MCD peptide amino acid sequence is Ile-Lys-Cys-Asn-Cys-Lys-Arg-His-Val-Ile-Lys-Pro-His-Ile-Cys-Arg-Lys-Ile-Cys-Gly-Lys-Asn [94,95]. The MCD peptide causes mast cell degranulation and histamine release at low concentrations leading to inhibition of potassium channels. Moreover, this peptide is responsible for the swelling and pain after a sting as well as for the allergic reaction [94,96].

### 3.4. Other Peptides of Bee Venom

The other peptides found in BV are contained only in a small percentage and their functions are relatively unknown [49,97]. Adolapin accounts for about 1% of the total BV and is noted for its anti-inflammatory and analgesic effects [98,99]. Secapin accounts for about 0.5% of the total BV and consists of 21 amino acids with a high proline composition and one disulfide bridge [69,97]. Tertiapin accounts for about 0.1% of the total BV and also consists of 21 amino acids with one disulfide bridge [97,100].

### 3.5. Phospholipase A_2_

PLA_2_ is the most important enzyme in BV, which constitutes about 10% of the dry venom itself. This enzyme catalyzes the hydrolysis of the sn-2 fatty acyl-ester bond of membrane glycero-3-phospholipids, resulting in varied biological effects. Hydrolysis of these compounds generates lysophospholipids. PLA_2_, as well as its hydrolysis products, also acts on the biological membranes. This enzyme in combination with MEL causes lysis of the cell membrane. Unsaturated fatty acids, the products of the hydrolysis of this enzyme, are precursors for the synthesis of inflammatory mediators (leukotrienes and prostaglandins). Also, this enzyme is the most important allergen of BV [101,102,103,104,105].

### 3.6. Other Enzymes of Bee Venom

In a small percentage, BV also contains PLB, which catalyzes the hydrolysis of phosphoglycerides [67,106]. The enzyme hyaluronidase breaks down hyaluronic acid in tissues and creates openings for venom entry [49,70,107].

## 4. The Therapeutic Properties of Bee Venom 

The origins of apitherapy date back to 6000 years ago in ancient Egyptian medicine. Moreover, ancient Greeks and Romans also used several bee products for medicinal purposes. The first written records on the use of BV as a therapeutic agent were found in ancient writings of Hippocrates, Aristotle, Pliny, and Galen. In the 19th century in Russian and Austrian medical journals, one could find descriptions of painful joint, rheumatism, neuralgia, and heart pain treatments using bee stings [45,46,108,109,110]. In folk medicine and especially in Oriental medicine, BV is used as a rheumatic and pain killer, as well as for lowering blood pressure and cholesterol levels. Furthermore, BV has been used against infectious diseases in the treatment of certain inflammations, and for general immunological resilience. The oldest, and the only possible, method of treatment with BV in folk medicine was a direct bee sting in the desired body area. Besides a direct bee sting, in Oriental medicine, acupuncture using BV was also used and was called apipuncture. After the development of processes that enable isolation of BV, the procedure was carried out by subcutaneously injecting venom into the affected area or into acupuncture points [12,109,110,111].

Today, a large number of scientific papers describe numerous applications of BV for therapeutic purposes, especially in anticancer treatment, in which its various components show a wide range of different beneficial activities. 

## 5. Anticancer Effects of Bee Venom and Its Components

### 5.1. Anticancer Effects of Bee Venom

Today, large numbers of studies are being conducted to explore the antitumor action of BV towards different types of cancers and the underlying mechanisms. The anticancer effect is mainly accredited to a basic polypeptide, MEL, that makes up about 50% of the dry BV. Havas [112] was one of the first who recorded the impact of BV on cancer cells. Afterward, Mufson and colleagues [113] reported that MEL can pass through a phospholipid bilayer, and thus display its ability. The relation between MEL and cell membranes caused impairment of the phospholipid’s acyl groups, higher sensitivity to phospholipid hydrolysis by phospholipase, and increased synthesis of prostaglandins from arachidonic acid released from phospholipids. Furthermore, McDonald et al. [114] examined BV’s anticancer property in a mortality study which involved 580 beekeepers. Beekeepers were identified through the obituaries published in three different US beekeeping industry journals between 1949 and 1978. Based on the obituaries, they established the cause of death of the beekeepers and made a comparison with the general population. Results showed a slightly lower incidence of death from cancer in beekeepers professionally exposed to BV during their working life compared to the rest of the population and a significantly lower death rate from lung cancer, while the mortality from other diseases was equal to the rest of the general population. The obtained results were among the first to suggest the possible anticancer potential of BV. After that, numerous studies showed anticancer properties of BV and its major component MEL [3,12,15,16,17,21,27,47,115,116,117].

### 5.2. Anticancer Effect of Melittin

Hait et al. [118] were the first to demonstrate an inhibiting potential of MEL in vitro. They showed that MEL, as an inhibitor of calmodulin, inhibits the growth and clonogenic capacity of human leukemia cells. Lee and Hait [119] have also observed an inhibitory impact of MEL on astrocytoma cell growth. Lazo et al. [120] noted a comparable mechanism of action of MEL as an inhibitor of calmodulin in leukemia cells. They also noted that MEL enhances bleomycin toxicity in human granulocyte macrophages and erythroid stem cell colonies [121]. Hait and Lee [122] noted that the cytotoxicity of MEL is proportional to the antagonistic effect of calmodulin. The aforementioned studies support the pharmacological role of calmodulin as a potential intracellular target of MEL antiproliferative activity.

Additionally, Killion and Dunn [123] showed that leukemia cells are more sensitive to MEL action compared to normal mouse spleen cells and bone marrow cells, reasoning that bone marrow cells have several binding sites on the membrane for carbohydrates, and these places disappear in the adult spleen cells, while they are almost absent after neoplastic changes that could make cancer cells more sensitive to the peptide. Zhu et al. [124] have reported that MEL does not prevent the growth of normal cells at a concentration that prevents the proliferation of cancer cells such as lung cancer cells. The observed cell response differences indicated an unalike activation of signaling pathways between normal and cancer cells. MEL has proven particularly effective in cultured cells that express high levels of the ras oncogene [125,126]. MEL also enhances the PLA_2_ activation in the ras oncogene-transformed cells resulting in its selective destruction. The results suggest that the enhanced activation of PLA_2_ by MEL could be the target of MEL’s cytotoxicity against cancer cells [12].

### 5.3. Anticancer Effects of Phospholipase A_2_

MEL causes increased activation of PLA_2_ activity and calcium intake in ras-transformed cells, which could be the basis for the antitumor activity of this compound [126]. Following these findings, a large number of studies made a connection between PLA_2_ activity and MEL’s cytotoxic effect on a variety of tumor cells [127,128,129,130]. Activation of PLA_2_ could play a role in the cytotoxicity of tumor cells through several different cell changes such as a synergistic effect of PLA_2_ and phosphatidylinositol (3,4)-bisphosphate in the induction of cell death [131]. Death caused by PLA_2_ and phosphatidylinositol (3,4)-bisphosphate is associated with the disruption of cell membrane integrity, abolition of signal transduction, and creation of a cytotoxic lyso-phosphatidylinositol (3,4)-bisphosphate. It was also found that their combined effect results in the formation of a tumor lysate that enhances the maturation of human monocyte-derived immunostimulatory dendritic cells. Such a tumor lysate, which is a complex mixture of tumor antigens with potential activity, has everything needed for a potential tumor vaccine [132].

### 5.4. The Mechanisms of Bee Venom and Melittin Anticancer Activity

One of the main issues in anticancer therapy is related to the concentration of the substance used, as it may cause serious side effects. Therefore, drug intake should be adequate and specific. A large number of insect lithic peptides, including those isolated from BV, have an amphipathic structure that binds and incorporates into negatively charged cell membranes. Compared to normal cells, which have a low membrane potential, the membrane of cancer cells has a high membrane potential [12,15,133,134] and that is why numerous lytic peptides selectively disrupt the membrane structure of cancer cells rather than the normal cell membrane. MEL should thus have a suitable role in anticancer therapy [12]. Gawronska et al. [135] have thus found that MEL is toxic to ovarian cancer cells and that the toxicity is dose-dependent.

Bee venom-induced apoptosis has been observed both in vitro and in vivo. Liu et al. [136] observed that BV inhibits the proliferation of melanoma cancer cells both in vitro and in vivo. The apoptosis observed in those cells was regarded as one of the possible mechanisms of action by which BV inhibits proliferation and induces differentiation of those same cells in vitro. Apoptosis was also observed in lung cancer cells by inhibition of cyclooxygenase 2 (COX-2) [137] and in osteosarcoma cells by increased Fas expression after BV treatment [138]. Holle et al. [134] observed that the MEL avidin conjugate has strong cytolytic activity in cells with a high metalloproteinase activity, such as prostate and ovary cancer cells. In contrast, the same activity was much lower in normal cells with limited metalloproteinase activity in vitro. In vivo, a significant reduction in tumor size was observed after treatment with the MEL avidin conjugate compared to untreated tumors. These studies also suggest the possible application of MEL avidin conjugate for therapeutic purposes. Moon et al. [133] suggested a molecular mechanism by which BV causes apoptosis in leukemia. Apoptosis was induced by reduced regulation of ERK and Akt signaling pathway. Furthermore, apoptosis induced by BV was associated with the downregulation of Bcl-2, caspase-3 activation, and cleavage of poly (ADP-ribose) polymerase (PARP). Moreover, induction of apoptosis was accompanied by a reduced regulation of inhibitory apoptosis protein (IAP proteins). BV also activated p38, MAPK, and JNK and decreased regulation of ERK and Akt [12].

These results indicate that the induction of apoptosis might have a role in the anticancer activity of BV and MEL, although the mechanisms behind this induction have still not been fully elucidated. Moreover, the apoptosis induction in cancer cells is also shown throughout gene therapy with MEL [139]. As the possibility of using the peptides from BV in anticancer therapy has been attracting increasing attention in recent years, Hu et al. [140] also found that these peptides could successfully kill liver cancer cells both in vitro and in vivo. A major mechanism of cancer growth inhibition by these peptides is again cell death induced by apoptosis. Oršolić et al. [141] have found that intravenous application of BV significantly reduces the number of lung metastases in mice. However, subcutaneous BV intake failed to show such a good effect on metastases, indicating route dependence as well as the proximity effects of BV when used for anticancer purposes.

Previous studies indicated that BV and MEL can induce strong toxic effects in various cancer cells such as lung, liver, kidney, breast, prostate, bladder, and leukemic cells, with a less pronounced effect in normal cells [12,15,16]. The proposed mechanisms of action are mainly related to the activation of PLA_2_, caspase, and matrix metalloproteinases that destroy cancer cells [133,134]. Conjugation of MEL with hormone receptors and MEL gene therapy could be useful in the future treatment of breast and prostate cancer [139,142,143,144]. Accordingly, MEL as an amphipathic protein may have a desirable role in therapeutic purposes. MEL is particularly active against cultured cells that express high levels of the ras oncogene [125,126]. Additionally, MEL enhances PLA_2_ activity in the ras oncogene-transformed cells, which results in their selective destruction, suggesting that such hyperactivation of PLA_2_ by MEL could be one of the major pathways of MEL’s cytotoxic activity against cancer cells [12].

In the past few decades, numerous studies showed quite potent anticancer effects of BV and MEL towards various cancer cells such as hepatocellular cells, prostate cells, lung cells, bladder cells, ovarian cells, mammary cells, and melanocyte cells, as well as in leukemia through different mechanisms of action [12,16,21,145].

**Figure 6:**
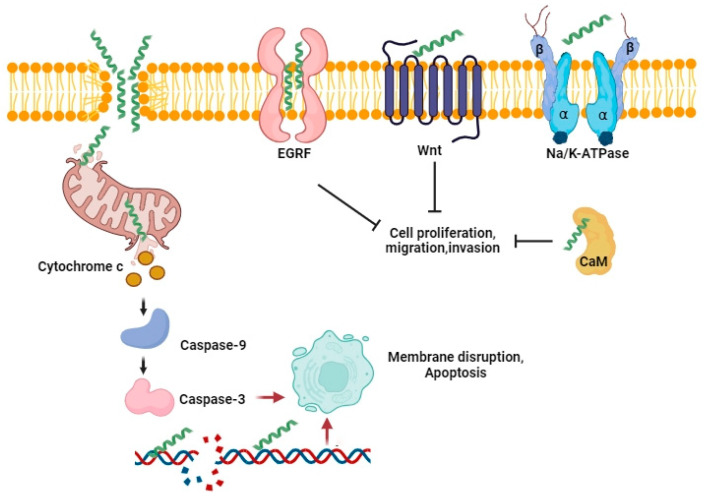
Simplified presentation of the mechanisms of melittin action. Melittin is presented as small helices. The peptide makes pores in the plasmatic membrane and destroys some of the membrane receptors and enzymes. Inside cells, melittin damages mitochondria and binds calmodulin, resulting in apoptosis and impairment of signaling pathways.

The numerous cellular effects of BV and MEL summarized above need explanation on a molecular level, and the main issue here has to do with the trigger of the apoptotic cascade. Apoptosis could be either a consequence of the plasmatic membrane fenestration or the result of the direct interaction of BV components with pro-apoptotic and anti-apoptotic factors. Interaction of BV peptides and enzymes with the plasma membrane is a crucial step in the whole process (Figure 6).

Application of biophysical methods showed that MEL brought a small decrease in local membrane fluidity in homogeneous lipid membranes, as the lipids appear to be more closely packed in the proximity of the MEL pore. On the contrary, in heterogeneous lipid membranes in cells, the local order of lipids is diminished by the peptide [146]. The selective affinity of MEL to cancer cells is determined mostly by acidic phosphatidylserine exposure to the outer layer of the cell membrane in cancer cells [147]. The binding of MEL to the membranes causes the formation of non-bilayer lipid phases in the membranes [148]. According to data from computer modeling, after penetration, the lipid bilayer MEL can adopt either a transmembrane or a U-shaped conformation. Several peptides of different conformations aggregate to form a pore. In the pores, peptides are organized in a manner such that polar residues face inward and hydrophobic residues face outward, which stabilizes the pores and forms water channels [149]. Depending on the local concentration of MEL, it can induce toroidal pores owing to the collective insertion of multiple MEL peptides from the N-termini. The pore formation is initiated by a local increase in membrane curvature in the vicinity of the peptide aggregate. Pore formation can be also achieved by a detergent-like mechanism when lipids are extracted or bursting, causing rapid formation of a large pore in a strongly curved membrane [150]. Membrane cholesterol impedes pore formation by MEL [151]. Membrane deformities induced by MEL enhance the activity of PLA_2_, and the synergistic action of the two BV components enhances the lytic effect of the venom [152]. 

Besides membrane lipids, MEL can directly interact with plasma membrane proteins, Na/K ATPase, for example. Binding causes inhibition of the enzyme [153]. MEL stimulates TRPM2 Ca^2+^ channels in glioblastoma cells, decreasing their resistance to chemotherapy [154]. BV and MEL suppress the activation of EGFR and HER2 in triple-negative and HER2-enriched breast cancer cells by interfering with the phosphorylation of these receptors in the plasma membrane [155]. Reports about the suppression of the Wnt/β-catenin pathway by MEL suggest the destruction of the Wnt receptors by the peptide [156]. 

What happens after the formation of the pore? Sure, it enables an influx of free radicals which can damage the cell; however, it seems that MEL action is better targeted. It was shown that MEL directly affected the mitochondrial membrane of the human lung adenocarcinoma cells A549. MEL caused changes in mitochondrial membrane potential, triggered mitochondrial ROS burst, and activated the mitochondria-related apoptosis pathway Bax/Bcl-2 [157]. Interaction with the mitochondrial membrane is localized to the cardiolipin-rich sites, where non-bilayer structures are formed [158]. Indeed, this effect is already sufficient to induce the terminal stage of apoptosis—leakage of cytochrome C, formation of apoptosomes, activation of executioner caspases, and fragmentation of chromatin. 

MEL can also interact with proteins involved in different regulation pathways. MEL and calmodulin complexes can even be crystallized [159] and used as a model system of protein–protein complexes. Multiple binding modes exist. Whereas the helical structure of MEL remains, the swapping of its salt bridges and partial unfolding of its C-terminal segment can occur. Different sets of residues can anchor at the hydrophobic pockets of calmodulin, which are considered the main recognition sites [160]. A block of calmodulin can cause disruptions of the PI3K/Akt and other pathways caused by BV; numerous data have been summarized in recent exhaustive reports [22,145]. 

Being a positively charged peptide, MEL can directly interact with DNA and RNA [161,162]. Data about these interactions are few and might indicate direct damage of DNA by MEL or interference in the transcription mechanism. Treatment with BV triggers the intensive accumulation of the γ-H2AX histone, a marker of the DNA double-strand breaks, in cancer cell nuclei, but the effect is not observed in normal fibroblasts [163]. MEL binds centrin, an enzyme involved in nucleotide-excision repair. Binding is stabilized by the hydrophobic triads—leucine–leucine–tryptophan [161]. BV causes changes in the mitochondrial genome by modification of the methylation pattern and mitochondrial DNA copy number [164].

## 6. Conclusions and Future Prospects

Since ancient times, it has been known that many natural compounds, herbs, and spices have different beneficial properties that are used to treat a variety of diseases, including cancer [32,34]. The term chemoprevention, established in the late 1970s, includes prevention and treatment of tumors by chemical compounds usually derived from plants. The research area of chemoprevention is studying such compounds to establish their potential effectiveness [165]. Using natural products as chemopreventive agents has increased dramatically in recent years and large numbers of such compounds are tested on different models. A large number of those chemicals and compounds, which provided promising results in experimental systems, are today already in pre-clinical studies, while for a large number of chemicals, science is still seeking the exact mechanism of action. Furthermore, numerous studies have shown that such chemicals used alone do not exhibit the desired results, but that their combination with existing chemotherapeutic agents may be promising, which is why this area has become particularly interesting to the scientific community [33,166,167]. 

Insects and their products have been used since ancient times in folk medicine to treat a variety of diseases. Numerous studies indicate that the use of those products, in addition to conventional treatments, could provide great benefits in combating many difficult but preventable diseases. Some promising therapies have already been experimentally tested [35,168]. Products that are obtained from bees, like honey, were used for the treatment of chronic and post-surgical wound and burn treatment, and in many cases have proven to be as effective as standard medicinal preparations. Moreover, beeswax is successfully used for the treatment of several dermatological disorders including psoriasis, dermatitis, numerous fungal skin diseases, and for skin discolorations. Royal jelly is used to treat postmenopausal symptoms, while propolis is used in the treatment of gastric ulcers [16,35,169,170]. In addition, BV is traditionally used as an anti-rheumatic, as a pain killer, for blood pressure treatment, and for lowering cholesterol levels. Furthermore, BV is used against infectious diseases, in the treatment of inflammations, and to improve general immunity [50,171,172,173].

Besides the traditional applications of BV, modern science has begun to research the possible anticancer potential of not only BV, but also of its components. BV toxicity is dependent on constituents such as MEL and PLA_2_, whose activity is amplified by MEL, as well as on several other small peptides such as apamin and MCD peptide [12,15,16,172,174]. Research on the medical applications of natural compounds derived from plants and animals is common not only in Oriental but also in Western medicine. Many of these studies deal with the mechanism of action of venoms and especially of BV on various cancer cells. A large number of currently available studies suggest that this toxin could have applications in the treatment of various cancers, but the exact mechanism of action of this anticancer effect has still not been fully understood. Some of the mechanisms of BV and MEL are related to the activation of PLA_2_, matrix metalloproteinases, and caspases that can destroy cancer cells [133,134]. Conjugation of MEL with hormone receptors and gene therapy with MEL could be used in the further development of cancer treatment [139,142,143,144]. Ling et al. [175] also noted an inhibitory effect towards carcinoma in vitro and in vivo of the recombinant virus carrying the MEL gene which could also be one way of combating cancers.

MEL is an especially interesting candidate in cancer therapy due to its lytic activity [176,177,178]. But apart from the toxic action of BV and MEL on cancer cells, its toxic effect on normal cells is also something that should be kept in mind. Studies have shown that BV and MEL display cytotoxic effects on cancer cells, but their cytotoxic activity towards normal non-cancerous cells is also expressed which makes them insufficiently suitable candidates for the development of new chemotherapeutic drugs [3,82,179,180,181,182,183,184,185]. What might be helpful in this case is finding a suitable carrier to transport MEL to the desired location. Incorporation of MEL in nanoparticles that possess the ability to carry a noteworthy amount of MEL to the cells of choice would be a useful way to suppress tumors and reduce melittin toxicity [186,187,188]. Additionally, there is a new genetically engineered vesicular antibody–melittin drug delivery platform that could be used for targeted cancer combination therapy [189]. Another option would be combination therapy that uses some of the existing cytostatic or other drugs of choice in combination with BV and/or MEL, where the additive/synergistic effect of the two agents may provide desirable results in the suppression of tumors, but could also lead to a reduction in the concentration of the existing cytostatic drug in the course of therapy, which could subsequently reduce undesirable effects caused by chemotherapy in many patients [190,191,192,193,194].

Up until now, research has shown a quite potent anticancer potential of both crude bee venom and MEL by inducing apoptosis and inhibiting the cell cycle without significantly affecting physiological cells. Moreover, increasing evidence from animal studies indicates the safety of venom doses effective in in vitro studies [47]. Studies done mainly on mice and rats using both whole BV and MEL indicated inhibition of tumors and metastasis growth, suppression of tumor proliferation, inhibition of angiogenesis, reduction of tumor size, and induction of apoptosis [195,196,197,198,199,200]. To the best of our knowledge, currently, there have been no clinical trials on humans that could confirm the clinical effectiveness of bee venom and evaluate the safety of its administration concerning cancer treatment, although there have been several clinical trials either completed or recruiting for other disorders which are registered at https://clinicaltrials.gov/ (Accessed on 5 February 2024).

As for now, the possibilities of clinical applications of BV as a sole drug are still distant, but the ongoing research on this topic could bring us closer to the possibility of using it in the future. Therefore, further research should focus on the cellular and molecular mechanisms of action of BV and its constituents on different cell types to determine their beneficial effects that could potentially be used in future anticancer therapy. However, before its possible clinical use, the route of injection, molecular target, mechanism of action, exact dosage, and possible side effects that they might have on normal cells and tissues, as well as other fundamental parameters, should be further investigated to avoid any possible adverse event. Making BV applicable requires extensive pre-clinical trials, with some applications also demanding clinical trials. 

## Figures and Tables

**Table 1:** Composition of dry bee venom (BV) expressed as type of molecule, components, and weight percentages.

Class of Molecules	Components	% of Dry BV
Enzymes	Phospolipase A_2_	10–12
	Hyaluronidase	1–3
	Acid phosphomonoesterase	1
	Lysophopholipase	1
	α-glucosidase	0.6
Proteins and peptides	Melittin	40–50
	Apamin	1–3
	Mast cell degranulating peptide	1–2
	Secapin	0.5–2
	Procamine	1–2
	Adolapin	1.0
	Protease inhibitor	0.8
	Tertiapin	0.1
	Other small peptides (<5 amino acids)	13–15
Physiologically active amines	Histamine	0.5–2.0
	Dopamine	0.2–1.0
	Noradrenalin	0.1–0.7
Amino acids	Aminobutyric acid	0.5
	α-amino acids	1
Sugars	Glucose and fructose	2
Phospholipids		5
Volatile compounds		4–8

## Data Availability

Not applicable.
